# Quartets enable statistically consistent estimation of cell lineage trees under an unbiased error and missingness model

**DOI:** 10.1186/s13015-023-00248-w

**Published:** 2023-12-01

**Authors:** Yunheng Han, Erin K. Molloy

**Affiliations:** 1 Department of Computer Science, University of Maryland, College Park, MD USA; 2 University of Maryland Institute for Advanced Computer Studies, College Park, MD USA

**Keywords:** Tumor phylogenetics, Cell lineage trees, Quartets, Supertrees, ASTRAL

## Abstract

Cancer progression and treatment can be informed by reconstructing its evolutionary history from tumor cells. Although many methods exist to estimate evolutionary trees (called phylogenies) from molecular sequences, traditional approaches assume the input data are error-free and the output tree is fully resolved. These assumptions are challenged in tumor phylogenetics because single-cell sequencing produces sparse, error-ridden data and because tumors evolve clonally. Here, we study the theoretical utility of methods based on quartets (four-leaf, unrooted phylogenetic trees) in light of these barriers. We consider a popular tumor phylogenetics model, in which mutations arise on a (highly unresolved) tree and then (unbiased) errors and missing values are introduced. Quartets are then implied by mutations present in two cells and absent from two cells. Our main result is that the most probable quartet identifies the unrooted model tree on four cells. This motivates seeking a tree such that the number of quartets shared between it and the input mutations is maximized. We prove an optimal solution to this problem is a consistent estimator of the unrooted cell lineage tree; this guarantee includes the case where the model tree is highly unresolved, with error defined as the number of false negative branches. Lastly, we outline how quartet-based methods might be employed when there are copy number aberrations and other challenges specific to tumor phylogenetics.

**Supplementary Information:**

The online version contains supplementary material available at 10.1186/s13015-023-00248-w.

## Introduction

Cancer progression and treatment can be informed by reconstructing the evolutionary history of tumor cells [1]. Although many methods exist to estimate evolutionary trees (called phylogenies) from molecular sequences, traditional approaches assume the input data are error-free and the output tree is fully resolved. These assumptions are challenged in tumor phylogenetics because single-cell sequencing produces sparse, error-ridden data and because tumors evolve clonally so the underlying tree is highly unresolved [2, 3]. Here, we study the theoretical utility of methods based on quartets (four-leaf, unrooted phylogenetic trees) and triplets (three-leaf rooted phylogenetic trees) in light of these barriers.

A *quartet* is an unrooted, phylogenetic tree with four leaves. Quartets have long been used as the building blocks for reconstructing the evolutionary history of species [4]. The reason quartet-based methods have garnered such success in species phylogenetics is their good statistical properties under the Multi-Species Coalescent ($\texttt {MSC}$) model [5, 6]. An $\texttt {MSC}$ model species tree generates gene trees (note that a gene tree reflects the genealogical history of a gene, which is passed down from ancestor to descendant, whereas the species tree governs the pool of potential ancestors). Arguably, one of the most important theoretical results from the last decade of systematics is that the most probable unrooted gene tree under the $\texttt {MSC}$ is topologically equivalent to the unrooted model species tree when considering four species [7]. For trees with more than four leaves, the most probable unrooted gene tree can be topologically discordant with the unrooted model species tree [8]. In such situations, the model species tree is said to be in the anomaly zone or the offending gene tree is said to be *anomalous*. It is now widely recognized that anomalous gene trees can challenge traditional species tree estimation methods [9, 10].

The statistical theory described above has motivated the development of quartet-based methods (e.g., [11, 12]) and is central to their proofs of statistical consistency under the MSC. ASTRAL [12], in particular, has become a gold standard approach to multi-locus species tree estimation. Moreover, new and improved quartet-based methods are continually being developed [13–18]. Similar theory and methodology has been given for *triplets*: three-leaf, rooted, phylogenetic trees [19–21].

Inspired by these efforts, we study the utility of quartets and triplets for estimating cell lineage trees under a popular tumor phylogenetics model [2, 22–24], in which mutations arise on a (highly unresolved) cell lineage tree according to the infinite sites model and then errors and missing values are introduced to the resulting mutation data in an unbiased fashion. The idea is that deviations from a perfect phylogeny can be attributed to sequencing errors, as data produced by single-cell protocols are notoriously error-prone and sparse. Although the infinite sites plus unbiased error and missingness ($\texttt {IS+UEM}$) model generates mutations rather than gene trees, quartets (or triplets) are implied by mutations that are present in two cells and absent from two cells (or one cell).

Our main result is that there are no anomalous quartets under the $\texttt {IS+UEM}$ model; this motivates seeking a cell lineage tree such that the number of quartets shared between it and the input mutations is maximized. We prove an optimal solution to this problem is a consistent estimator of the unrooted cell lineage tree; this guarantee extends to the case of highly unresolved model trees, with error defined as the number of false negative branches. Somewhat surprisingly, our positive finding for quartets does not extend to triplets, as there can be anomalous triplets under the $\texttt {IS+UEM}$ model under reasonable conditions. These results generalize to any model of 2-state character evolution for which there are no anomalous quartets or triplets. An example of such a model is the infinite sites plus neutral Wright-Fisher ($\texttt {IS+nWF}$) model [25, 26] and its approximations [46]. Under $\texttt {IS+nWF}$, mutations follow the IS assumption but evolve within a species tree, so deviations from a perfect phylogeny are due to genetic drift. Nevertheless, there are no anomalous triplets (see Additional file 1 of [47]) and no anomalous quartets (Theorem 1 in [48]; also see [49]), motivating the application of quartet-based methods to estimate species trees from low-homoplasy retroelement insertion presence/absence patterns [48, 50]. However, our work is largely motivated by tumor phylogenetics, so we conclude by outlining how quartet-based methods might be employed in this setting, given other important challenges like copy number aberrations (CNAs) and doublets.

## Background

We now provide some background on phylogenetic trees, models of evolution, and statistical consistency.

### Phylogenetic trees

A *phylogenetic tree* is defined by the triple $(g, X, \phi )$, where *g* is a connected acyclic graph, *X* is a set of labels (often representing species or cells), and $\phi$ is a bijection between the labels in *X* and leaves (i.e., vertices with degree 1) of *g*. Phylogenetic trees can be either *unrooted* or *rooted*, and we use *u*(*T*) to denote the unrooted version of a rooted tree *T*. Edges in an unrooted tree are undirected, whereas edges in a rooted tree are directed away from the root, a special vertex with in-degree 0 (all other vertices have in-degree 1). Vertices that are neither leaves nor the root are called internal vertices, and edges incident to only internal vertices are called *internal edges* (otherwise they are referred to as *terminal* edges). An interval vertex with degree greater than 3 (called a *polytomy*) can be introduced to a tree by contracting one of its edges (i.e., deleting the edge and identifying its endpoints). A *refinement* of a polytomy is the opposite of a *contraction*. If there are no polytomies in *T*, we say that *T* is *binary* or *fully resolved*; otherwise, we say that *T* is *non-binary*. We use the phrase *highly unresolved* to indicate that *T* contains many polytomies and/or that the polytomies in *T* have high degrees.

As previously mentioned, many methods for species tree estimation are based on *quartets*. A quartet is an unrooted, binary tree with four leaves. We denote the three possible quartets on $X = \{A, B, C, D\}$ as $q_1 = A,B|C,D$, $q_2 = A,C|B,D$, and $q_3 = A,D|B,C$. A set of quartets can be created from an unrooted tree *T* by restricting *T* to every possible subset of four leaves (i.e., deleting the other leaves from *T* and then suppressing vertices of degree 2). The resulting set *Q*(*T*) is called the quartet encoding of *T*, and we say that *T* displays quartet *q* if $q \in Q(T)$. Importantly, if *T* contains polytomies, restricting *T* to some subsets of four labels will not produce a (binary) quartet. Some selections will produce star trees, which do not provide any topological information. We use $T|_S$ to denote *T* restricted to label set *S* (note that if branch parameters are associated with *T*, they are added together when suppressing vertices of degree 2). The concepts described above for quartets extend to triplets. A *triplet* is a rooted, binary tree with three leaves, and we denote the three possible triplets on $X = \{A, B, C\}$ as $t_A = A|B,C$, $t_B = B|A,C$, and $t_C = C|A,B$. Lastly, a *bipartition* or *split* of label set *X* partitions it into two disjoint subsets. It is easy to see that each edge in an unrooted tree induces a bipartition, and we use *Bip*(*T*) to denote the set of bipartitions induced by all edges in *T*.

### Mutations and models of evolution

A mutation matrix *M* is an $n \times k$ matrix, where *n* is the number of rows (representing cells or species) and *k* is the number of columns (representing mutations). Columns are also referred to as characters or site patterns. Our focus here is on 2-state characters, with $M_{i,j} = 0$ indicating that mutation *j* is absent from cell *i* and $M_{i,j} = 1$ indicating that mutation *j* is present in cell *i*. In tumor phylogenetics, mutations are called in reference to a healthy cell, which is the root of the cell lineage tree; thus, 0 represents the ancestral state and 1 represents mutant/derived state (note that this interpretation of states 0 and 1 will only be important when looking at triplets and not quartets).

**Fig. 1:**
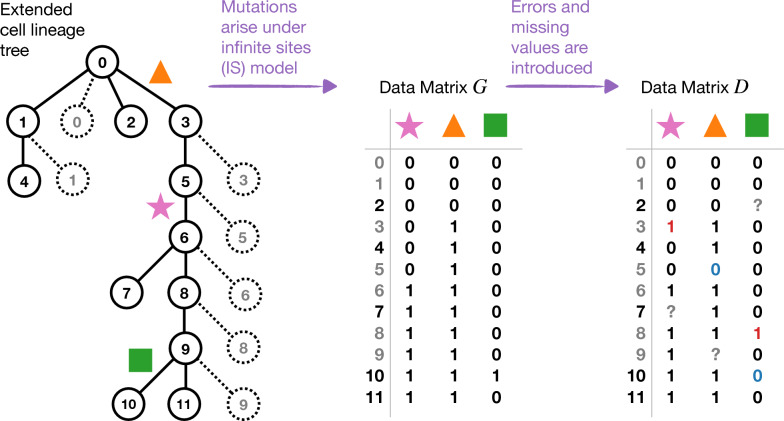
The schematic shows a model cell lineage tree, where the dashed lines and circles are “fake” edges and vertices, respectively. If we assume a mutation occurs on any non-fake edges with equal probability (as in [24]), then the probability of a mutation on any solid edge will be 1/11. Mutations cannot occur on any of the dashed edges. Data are generated from this model cell lineage tree in two steps. First, mutations arise on the tree under the IS model, producing data matrix *G*. Second, false positives (0 flips to 1; shown in red), false negatives (1 flips to 0; shown in blue), and missing values (0/1 flips to ?; shown in grey) are introduced to G under the UEM model, producing data matrix *D*

Throughout this paper, we assume the mutation matrix *D* is generated under a *hierarchical model* with two steps (Fig. 1).

1. A mutation matrix *G* is generated under some model ${\mathcal {M}}$, parameterized by a rooted phylogenetic tree topology $\sigma$ and a set $\Theta$ of associated numeric parameters. Importantly, model ${\mathcal {M}}$ given $(\sigma , \Theta )$ defines a probability distribution on mutation patterns, and we assume mutations in *G* are *independent and identically distributed (i.i.d.)* according to this model. For simplicity of notation, we typically omit the dependence on the numeric parameters in $\Theta$.
2. Errors and/or missing values are introduced to the ground truth matrix *G* according to the UEM model (described below). This result of this process is the observed matrix *D*.

Hierarchical models of this form, denoted ${\mathcal {M}}{} \texttt {+UEM}$, define a probability distribution on mutation patterns given their parameters. Thus, if we say that *D* is generated under the ${\mathcal {M}}{} \texttt {+UEM}$ model, then we assume the mutations in *D* are *i.i.d.* according to this model. We now describe the data generation steps in greater detail for a popular tumor phylogenetics model [2, 22–24].

*Step 1: Infinite Sites* (IS) *model.* For tumor phylogenetics, we take ${\mathcal {M}}$ to be the infinite sites (IS) model, so the mutation matrix *G* is generated under the IS model given a rooted cell lineage tree $\sigma$ and a set $\Theta$ of edge probabilities that sum to 1. Specifically, every edge *e* in $\sigma$ is associated with a numeric value $p(e) \in \Theta$, indicating the probability that a mutation occurs on *e*. When a mutation occurs on *e*, all cells on a directed path from *e* to any of the leaves of $\sigma$ are set to state 1; all other cells are set to state 0. Thus, a mutation corresponds to the bipartition induced by the branch on which it occurred. Internal edges on which mutations cannot occur are contracted, so that the probability of a mutation on any edge in $\sigma$ is strictly greater than zero. Terminal edges on which mutations cannot occur are not contracted; however, we refer to these edges and (the leaves incident to them) as “fake”.

*Step 2: Unbiased Error and Missingness* (UEM) *model.* If mutation matrix *G* is generated under the IS model given $(\sigma , \Theta )$, then reconstructing $\sigma$ is trivial. However, for tumor phylogenetics, false positives and false negatives are introduced to *G*, producing the observed matrix *D*. This is done according to Eq. 1:

1 $$\begin{aligned} \mathbb {P}(D_{i,j} = x | \alpha , \beta , G_{i,j} = y) = {\left\{ \begin{array}{ll} (1 - \alpha ) &{}\text { if }\, D_{i,j} = 0 \text { and }\, G_{i,j} = 0 \\ { \alpha } &{}\text { if }\, D_{i,j} = 1 \text { and }\, G_{i,j} = 0 \\ { \beta } &{}\text { if }\, D_{i,j} = 0 \text { and }\, G_{i,j} = 1 \\ (1 - \beta ) &{}\text { if }\, D_{i,j} = 1 \text { and }\, G_{i,j} = 1 \\ \end{array}\right. } \end{aligned}$$

where $0 \le \alpha < 1$ and $0 \le \beta < 1$ are the probability of false positives and false negatives, respectively. Simultaneously, missing values are introduced to *G* with probability $0 \le \gamma < 1$; this can be incorporated into the model by multiplying each of the cases in Eq. 1 by $(1 - \gamma )$.

Our goal is to estimate cell lineage trees under the $\texttt {IS+UEM}$ model. An important property for phylogeny estimation methods is whether they are statistically consistent under the model of interest.

#### Definition 1

(Statistical Consistency; see Section 1.1 of [27]) Let ${\mathcal {A}}$ be some model that generates mutations, and let *D* be a mutation matrix, with *n* rows (cells or species) and *k* columns (mutations), generated under ${\mathcal {A}}$ given rooted tree $\sigma$ and numerical parameters $\Theta$. We say that an estimation method is *statistically consistent* under ${\mathcal {A}}$ if for any $\epsilon > 0$, there exists a constant $K > 0$ such that when *D* contains at least *K* mutations, the method given *D* returns (the unrooted version of) $\sigma$ with probability at least $1-\epsilon$. Alternatively, we might say that the error in the tree estimated from *D* is zero with probability at least $1-\epsilon$.

The idea is that as the number *k* of mutations goes towards infinity, the error in the estimated tree is zero with high probability. Tree error is typically defined as the number of *false negative branches* (i.e., branches in $\sigma$ that are missing from the estimated tree) plus the number of *false positive branches* (i.e., branches in the estimated tree that are missing from $\sigma$).

## No anomalous quartets under an unbiased error and missingness model

**Fig. 2:**
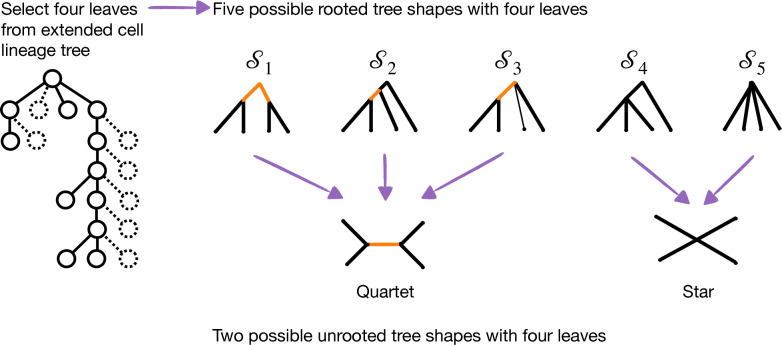
There are five possible tree shapes with four leaves. Three of these tree shapes (${\mathcal {S}}_1$, ${\mathcal {S}}_2$, and ${\mathcal {S}}_3$) have a non-trivial unrooted topology (quartet). The other two tree shapes (${\mathcal {S}}_4$ and ${\mathcal {S}}_5$) have a trivial unrooted topology (star). Looking at Fig. 1, tree shapes ${\mathcal {S}}_1$–${\mathcal {S}}_5$ are observed by sampling cells $X_1 = \{1, 4, 10, 11\}$, $X_2 = \{1, 7, 10, 11\}$, $X_3 = \{1, 2, 10, 11\}$, $X_4 = \{1, 9, 10, 11\}$, and $X_5 = \{0, 1, 2, 3\}$, respectively

To begin, we assume that the rooted cell lineage tree $\sigma$ has four leaves; therefore, it must have one of five tree shapes shown in Fig. 2. Two of them display a star when unrooted, and the other three correspond to a quartet when unrooted. If mutations are generated *i.i.d.* under some model ${\mathcal {A}}$ given $\sigma$, there are 16 possible patterns on four cells, denoted $\{A, B, C, D\}$. A quartet is implied by two cells being in state 1 and two cells being in state 0. Therefore, two patterns ($ABCD = 0011$ and 1100) support quartet $q_1 = A,B|C,D$, two patterns (0101 and 1010) support quartet $q_2 = A,C|B,D$, two patterns (0110 and 1001) support quartet $q_3 = A,D|B,C$, and the other 10 patterns do not provide topological information. Henceforth, we denote the probability of quartets under model ${\mathcal {A}}$ given $\sigma$ as $\mathbb {P}_{{\mathcal {A}}}(q_1 | \sigma ) = \mathbb {P}_{{\mathcal {A}}}(1100 | \sigma ) + \mathbb {P}_{{\mathcal {A}}}(0011 | \sigma )$, $\mathbb {P}_{{\mathcal {A}}}(q_2 | \sigma ) = \mathbb {P}_{{\mathcal {A}}}(1010 | \sigma ) + \mathbb {P}_{{\mathcal {A}}}(0101 | \sigma )$, and $\mathbb {P}_{{\mathcal {A}}}(q_3 | \sigma ) = \mathbb {P}_{{\mathcal {A}}}(1001 | \sigma ) + \mathbb {P}_{{\mathcal {A}}}(0110 | \sigma )$. Now we consider quartet-informative patterns generated from a model tree with more than four leaves.

### Definition 2

(No anomalous quartets) We say that there are no anomalous quartets under model ${\mathcal {A}}$ given rooted tree $\sigma$ if the following inequalities hold for every subset *S* of four species in $\sigma$. Let $q_1, q_2, q_3$ denote the three quartets on *S*, and let *i* index $\{1, 2, 3\}$.

1. If $u(\sigma )|_S = q_i$, $\mathbb {P}_{{\mathcal {A}}}(q_i|\sigma ) > \mathbb {P}_{{\mathcal {A}}}(q_j|\sigma )$ for all $j \in \{1, 2, 3\}$ such that $i \ne j$.
2. If $u(\sigma )|_S$ is a star, $\mathbb {P}_{{\mathcal {A}}}(q_1|\sigma ) = \mathbb {P}_{{\mathcal {A}}}(q_2|\sigma ) = \mathbb {P}_{{\mathcal {A}}}(q_3|\sigma )$.

This brings us to the main result of this section.

### Theorem 1

There are no anomalous quartets under the $\texttt {IS+UEM}$ model, assuming $\alpha + \beta \ne 1$.

The statement above directly follows from Lemma 1 and Corollary 1.

### Lemma 1

There are no anomalous quartets under the $\texttt {IS}$ model. Moreover, all quartet-informative patterns have zero probability except for one or both of the patterns corresponding to $u(\sigma )$ when $u(\sigma )$ is not a star.

If $\sigma$ has more than four leaves, we can restrict $\sigma$ to any subset of four leaves and get a valid sub-model (i.e., a sub-model for which the probability of the mutation patterns on four cells is the same as under the larger model tree). For the IS model, the sub-model is formed by deleting the other leaves and adding branch parameters together when suppressing vertices of degree 2. The mutation pattern probabilities for the four cells under this sub-model will be the same as the larger tree because addition represents an *or* condition (i.e., a mutation occurring on this branch *or* on that branch will produce the same pattern when looking at only a subset of cells). Thus, it suffices to verify that there are no anomalous quartets for $\sigma$ with four leaves. This can be done by considering a mutation occurring on each of the internal branches of all possible rooted tree shapes with four leaves (Fig. 2) and comparing the resulting pattern to the unrooted tree shape; see Additional file 1 for details.

The following two lemmas will also be useful later.

### Lemma 2

Let $0 \le \alpha < 1$ and $0 \le \beta < 1$. Then,

2 $$\begin{aligned} \big ( (1 - \beta )^2 (1 - \alpha )^2 + \beta ^2 \alpha ^2 \big ) - 2 \beta (1 - \beta ) \alpha (1 - \alpha ) = \big ( 1 - (\alpha + \beta ) \big )^2 > 0 \end{aligned}$$

for $\alpha + \beta \ne 1$. If $\alpha + \beta = 1$, the inequality in Eq. 2 becomes an equality.

The statement above follows from expanding the polynomials; see the Additional file 1 for details.

### Lemma 3

If there are no anomalous quartets under model ${\mathcal {M}}$, then there are no anomalous quartets under the ${\mathcal {M}}{+}\texttt {UE}$ model, assuming $\alpha + \beta \ne 1$.

### Proof

Taking any subset of four leaves, there are 16 possible mutation patterns that may occur under model ${\mathcal {M}}$. These are the two invariant patterns (0000 and 1111), the eight variant but quartet-uninformative patterns (1000, 0100, 0010, 0001, 0111, 1011, 1101, 1110), and the six quartet-informative patterns (1100, 0011, 0101, 1010, 0110, 1001). For each pattern *g* listed above, we enumerate all possible ways of introducing errors (false positives and false negatives); this gives us the probability of each of the 16 mutation patterns under the $\texttt {UE}$ model given $(\alpha , \beta )$. Now we need to put this information together to get the probability of quartets under the ${\mathcal {M}}{} \texttt {+UE}$ model. First, we compute the probability of observing any quartet *q* from errors (false positives and false negatives) being introduced to the invariant and variant but quartet-uninformative characters; see Eq. 3.

3 $$\begin{aligned} f(\alpha , \beta , \sigma )&= \big ( 2 \alpha ^2 (1 - \alpha )^2 \big ) \cdot \mathbb {P}_{{\mathcal {M}}}(0000 | \sigma ) + \big ( 2 \beta ^2 (1 - \beta )^2 \big ) \cdot \mathbb {P}_{{\mathcal {M}}}(1111 | \sigma ) \nonumber \\&+ \bigg ( \big ( \alpha (1 - \alpha )^2 (1 - \beta ) \big ) + \big ( \alpha ^2 (1 - \alpha ) \beta \big ) \bigg ) \nonumber \\&\cdot \big ( \mathbb {P}_{{\mathcal {M}}}(0001 | \sigma ) + \mathbb {P}_{{\mathcal {M}}}(0010 | \sigma ) + \mathbb {P}_{{\mathcal {M}}}(0100 | \sigma ) + \mathbb {P}_{{\mathcal {M}}}(1000 | \sigma ) \big ) \nonumber \\&+ \bigg ( \big (\beta (1 - \beta )^2 (1 - \alpha ) \big ) + \big ( \beta ^2 (1 - \beta ) \alpha \big ) \bigg ) \nonumber \\&\cdot (\mathbb {P}_{{\mathcal {M}}}(1110 | \sigma )+ \mathbb {P}_{{\mathcal {M}}}(1101 | \sigma )+ \mathbb {P}_{{\mathcal {M}}}(1011 | \sigma )+ \mathbb {P}_{{\mathcal {M}}}(0111 | \sigma )); \end{aligned}$$

see Additional file 1: Tables S1–S4 for details. Second, we repeat this calculation for the quartet-informative patterns; see Table 1 and Additional file 1: Tables S5–S6 for details. Putting it all together gives us the probability of each quartet under the ${\mathcal {M}}{} \texttt {+UE}$ model

4 $$\begin{aligned} \mathbb {P}_{{\mathcal {M}}{} \texttt {+UE}}(q_i |\alpha , \beta , \sigma )&= f(\alpha , \beta , \sigma ) \nonumber \\&+ \big ((1-\alpha )^2(1-\beta )^2+\alpha ^2\beta ^2\big ) \cdot \mathbb {P}_{{\mathcal {M}}}(q_i|\sigma ) \nonumber \\&+ 2\alpha \beta (1-\alpha )(1-\beta ) \cdot \big (\mathbb {P}_{{\mathcal {M}}}(q_j|\sigma ) +\mathbb {P}_{{\mathcal {M}}}(q_k|\sigma ) \big ) \end{aligned}$$

for $i, j, k \in \{1, 2, 3\}$ such that $i \ne j \ne k$. Now we can compute the difference in probabilities between quartets $q_i$ and $q_j$ under the ${\mathcal {M}}{} \texttt {+UE}$ model for any $i,j \in \{1, 2, 3\}$ such that $i \ne j$. By Lemma 1, we have

5 $$\begin{aligned} \mathbb {P}_{{\mathcal {M}}{} \texttt {+UE}}(q_i |\alpha , \beta , \sigma )&- \mathbb {P}_{{\mathcal {M}}{} \texttt {+UE}}(q_j |\alpha , \beta , \sigma ) =\big ( 1 - (\alpha + \beta )\big )^2 \cdot \big ( \mathbb {P}_{{\mathcal {M}}}(q_i|\sigma ) - \mathbb {P}_{{\mathcal {M}}}(q_j|\sigma ) \big ). \end{aligned}$$

Assuming $\alpha + \beta \ne 1$, this quantity is zero if $\mathbb {P}_{{\mathcal {M}}}(q_i|\sigma ) = \mathbb {P}_{{\mathcal {M}}}(q_j|\sigma )$ and greater than zero if $\mathbb {P}_{{\mathcal {M}}}(q_i|\sigma ) > \mathbb {P}_{{\mathcal {M}}}(q_j|\sigma )$. Because there are no anomalous quartets under model ${\mathcal {M}}$, the former will be the case if $u(\sigma )$ is a star; the latter will be the case if $u(\sigma ) = q_i$. It follows there are no anomalous quartets under the ${\mathcal {M}}{} \texttt {+UE}$ model. $\square$

**Table 1:**
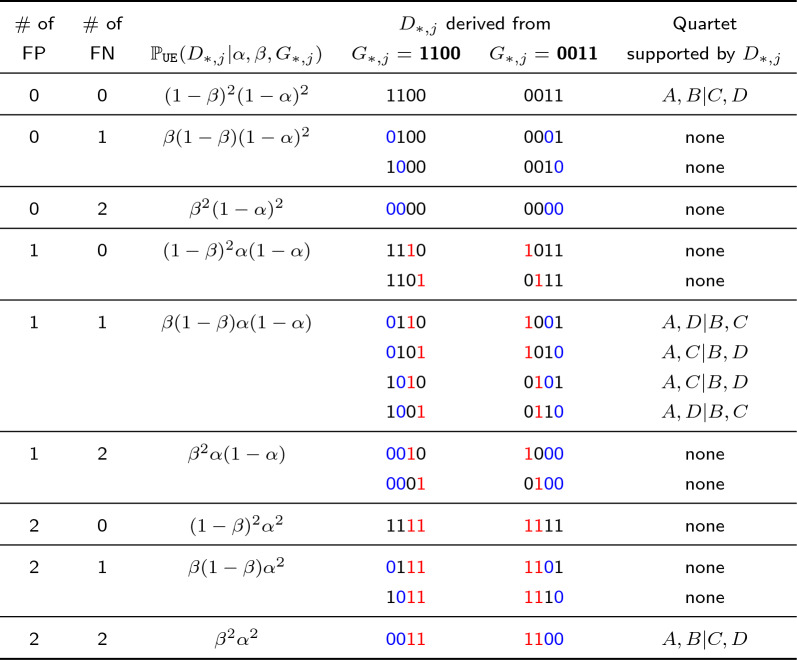
List of mutation patterns with four cells $\{A, B, C, D\}$ that can be generated by introducing false positives and false negatives to pattern #12 ($G_{*,j} = 1100$) and pattern #3 ($G_{*,j} = 0011$) as well as their probabilities under the $\texttt {UE}$ model

The red values indicate a false positive introduced to $G_{*,j}$ by flipping 0 to 1. The blue values indicate a false negative introduced to $G_{*,j}$ by flipping 1 to 0. Similar tables for the other 14 patterns are provided in Additional file 1

Note that the quantity $\alpha + \beta$ is unlikely to equal 1 in practice, as both probabilities should be less than 0.5. We now extend the result above to address unbiased missing values, in addition to errors.

### Corollary 1

If there are no anomalous quartets under model ${\mathcal {M}}$, then there are no anomalous quartets under the ${\mathcal {M}}{} \texttt {+UEM}$ model, assuming that $\alpha + \beta \ne 1$.

### Proof

If one or more of the values in a mutation pattern is missing, then no quartet is displayed. Thus, unbiased missingness can be accounted for in the proof of Lemma 3 simply by updating $\mathbb {P}_{{\mathcal {M}}}(x|\sigma )$ to $(1-\gamma )^4 \cdot \mathbb {P}_{{\mathcal {M}}}(x|\sigma )$. In this case, Eq. 5 becomes

6 $$\begin{aligned} \mathbb {P}_{{\mathcal {M}}{} \texttt {+UEM}}(q_i |\alpha , \beta , \gamma , \sigma )&- \mathbb {P}_{{\mathcal {M}}{} \texttt {+UEM}}(q_j |\alpha , \beta , \gamma , \sigma ) \nonumber \\&=\big ( 1 - (\alpha + \beta )\big )^2 \cdot (1 - \gamma )^4 \cdot \big ( \mathbb {P}_{{\mathcal {M}}}(q_i|\sigma ) - \mathbb {P}_{{\mathcal {M}}}(q_j|\sigma ) \big ) \end{aligned}$$

which does not change our argument. $\square$

This concludes the derivation of the main result of this section (Theorem 1). Before moving on to triplets, we note that the difference in quartet probabilities (Eq. 6) depends on (1) the probability of false positives and negatives (specifically how close their sum is to one), (2) the probability of missing values, and (3) the probability of observing quartet-informative patterns under model ${\mathcal {M}}$ given $\sigma$. In the simulations performed by [24], the largest values of $\alpha$, $\beta$, and $\gamma$ were 0.001, 0.2, and 0.05, respectively. In this scenario with $u(\sigma ) = q_i$, we have $\mathbb {P}_{\texttt {IS+UEM}}(q_i|\alpha , \beta , \gamma , \sigma ) - \mathbb {P}_{\texttt {IS+UEM}}(q_j|\alpha , \beta , \gamma , \sigma ) = 0.52 \cdot \mathbb {P}_{\texttt {IS}}(q_i | \sigma )$ for any $i, j \in \{1, 2, 3\}$ such that $i \ne j$. The magnitude of $\mathbb {P}_{\texttt {IS}}(q_i | \sigma )$ depends on the lineage tree and the four cells sampled from it. We discuss sampling further in the context of triplets; for now, we note that, in practical settings, $\mathbb {P}_{\texttt {IS}}(q_i | \sigma )$ may have a greater impact on the difference in quartet probabilities under the $\texttt {IS+UEM}$ model than $\alpha$, $\beta$, or $\gamma$.

## Anomalous triplets under an unbiased error and missingness model

We now derive related results for triplets. To begin, we assume the rooted cell lineage tree $\sigma$ has three leaves; therefore, it must have one of two topologies: binary or non-binary (Additional File 1: Fig S1). If mutations are generated *i.i.d.* under some model ${\mathcal {A}}$ given $\sigma$, there are 8 possible patterns on three cells, denoted $\{A, B, C\}$. A triplet is implied by two cells being in state 1 (i.e., the mutant/derived state) and one cell being in state 0 (i.e., the ancestral state) because the two cells harboring the mutation must have descended from a common ancestor cell also harboring the mutation. One pattern ($ABC = 110$) supports triplet $t_C = C|A,B$, one pattern (101) supports triplet $t_B = B|A,C$, one pattern (011) supports triplet $t_A = A|B,C$, and the other five patterns do not provide topological information. Henceforth, we denote the probability of triplets under model ${\mathcal {A}}$ given $\sigma$ as $\mathbb {P}_{{\mathcal {A}}}(t_A|\sigma ) = \mathbb {P}_{{\mathcal {A}}}(011|\sigma )$, $\mathbb {P}_{{\mathcal {A}}}(t_B|\sigma ) = \mathbb {P}_{{\mathcal {A}}}(101|\sigma )$, and $\mathbb {P}_{{\mathcal {A}}}(t_C|\sigma ) = \mathbb {P}_{{\mathcal {A}}}(110|\sigma )$. Now we consider triplet-informative patterns generated from a model tree with more than three leaves.

### Definition 3

(No anomalous triplets) We say that there are no anomalous triplets under model ${\mathcal {A}}$ if the following inequalities hold for every subset *S = {X, Y, Z}* of three species in $\sigma$. Let $t_X, t_Y, t_Z$ denote the three triplets on *S*, and let *i* index {*X*, *Y*, *Z*}.

1. If $\sigma |_S = t_i$, $\mathbb {P}_{{\mathcal {A}}}(t_i|\sigma ) > \mathbb {P}_{{\mathcal {A}}}(t_j|\sigma )$ for all $j \in \{X, Y, Z\}$ such that $i \ne j$.
2. If $\sigma |_S$ is non-binary, $\mathbb {P}_{{\mathcal {A}}}(t_X|\sigma ) = \mathbb {P}_{{\mathcal {A}}}(t_Y|\sigma ) = \mathbb {P}_{{\mathcal {A}}}(t_Z|\sigma )$.

This brings us to the main result of this section.

### Theorem 2

There are no anomalous triplets under the $\texttt {IS+UEM}$ model, assuming one of two conditions: (1) $\alpha = 0$ or (2) $\alpha + \beta \ne 1$ and $\mathbb {P}_{\texttt {IS}}(100|\sigma ) = \mathbb {P}_{\texttt {IS}}(010|\sigma ) = \mathbb {P}_{\texttt {IS}}(001|\sigma )$. Otherwise, there can be anomalous triplets under the $\texttt {IS+UEM}$ model.

The statement above directly follows from Lemma 4 and Corollary 2.

### Lemma 4

There are no anomalous triplets under the $\texttt {IS}$ model. Moreover, all triplet-informative patterns have zero probability except for the pattern corresponding to $\sigma$ when $\sigma$ is not non-binary.

If $\sigma$ has more than three leaves, we can restrict $\sigma$ to any subset of three leaves and get a valid sub-model (i.e., a sub-model for which the probability of the mutation patterns on three cells is the same as under the larger model tree, as discussed for quartets). Thus, it suffices to verify that there are no anomalous triplets for $\sigma$ with three leaves. This can be done by considering a mutation occurring on each of the internal branches of all possible rooted tree shapes with three leaves (Additional File 1: Fig S1) and comparing the resulting pattern to the tree shape; see Additional File 1 for details.

### Lemma 5

If there are no anomalous triplets under model ${\mathcal {M}}$, then there are no anomalous triplets under the ${\mathcal {M}}{} \texttt {+UE}$ model, assuming one of two conditions: (1) $\alpha = 0$ or (2) $\alpha + \beta \ne 1$ and $\mathbb {P}_{{\mathcal {M}}}(100|\sigma ) = \mathbb {P}_{{\mathcal {M}}}(010|\sigma ) = \mathbb {P}_{{\mathcal {M}}}(001|\sigma )$. Otherwise, there can be anomalous triplets under the ${\mathcal {M}}{} \texttt {+UE}$ model.

### Proof

Taking any subset of three leaves, there are 8 possible mutation patterns that may occur under model ${\mathcal {M}}$. These are the two invariant patterns ($ABC = 000$ and 111), the three variant but triplet-uninformative patterns (100, 010, 001), and the three triplet-informative patterns (110, 101, 011). For each pattern *g* listed above, we enumerate all possible ways of introducing errors (false positives and false negatives); this gives us the probability of mutation patterns under the UE model given $(\alpha , \beta , g)$; see Additional file 1: Tables S7–S14. Putting everything together, we find the probability of triplet $t_C$ under the ${\mathcal {M}}{} \texttt {+UE}$ model is

7 $$\begin{aligned} \mathbb {P}_{{\mathcal {M}}{} \texttt {+UE}}(t_C|\alpha ,\beta ,\sigma )&= \alpha ^2 (1 - \alpha ) \cdot \mathbb {P}_{{\mathcal {M}}}(000|\sigma ) + \beta (1 - \beta )^2 \cdot \mathbb {P}_{{\mathcal {M}}}(111|\sigma ) \nonumber \\&+ \alpha ^2\beta \cdot \mathbb {P}_{{\mathcal {M}}}(001|\sigma ) \nonumber \\&+ \alpha (1-\alpha )(1-\beta ) \cdot \big ( \mathbb {P}_{{\mathcal {M}}}(100|\sigma ) + \mathbb {P}_{{\mathcal {M}}}(010|\sigma )\big ) \nonumber \\&+ (1-\alpha )(1-\beta )^2 \cdot \mathbb {P}_{{\mathcal {M}}}(110|\sigma ) \nonumber \\&+ \alpha \beta (1-\beta ) \cdot \big ( \mathbb {P}_{{\mathcal {M}}}(101|\sigma ) + \mathbb {P}_{{\mathcal {M}}}(011|\sigma ) \big ) \end{aligned}$$

Similar probabilities can be computed for $t_B$ and $t_A$. To provide a general formula, we define

8 $$\begin{aligned} g(\alpha , \beta , \sigma ) = \alpha ^2 (1 - \alpha ) \cdot \mathbb {P}_{{\mathcal {M}}}(000|\sigma ) + \beta (1 - \beta )^2 \cdot \mathbb {P}_{{\mathcal {M}}}(111|\sigma ) \end{aligned}$$

and set $x_A = 100$, $x_B = 010$, and $x_C = 001$. This allows us to write the probability of any triplet as

9 $$\begin{aligned} \mathbb {P}_{{\mathcal {M}}{} \texttt {+UE}}(t_i|\alpha ,\beta ,\sigma )&= g(\alpha , \beta , \sigma ) \nonumber \\&+ \alpha ^2\beta \cdot \mathbb {P}_{{\mathcal {M}}}(x_i |\sigma ) \nonumber \\&+ \alpha (1-\alpha )(1-\beta ) \cdot \big ( \mathbb {P}_{{\mathcal {M}}}(x_j|\sigma ) + \mathbb {P}_{{\mathcal {M}}}(x_k|\sigma )\big ) \nonumber \\&+ (1-\alpha )(1-\beta )^2 \cdot \mathbb {P}_{{\mathcal {M}}}(t_i|\sigma ) \nonumber \\&+ \alpha \beta (1-\beta ) \cdot \big ( \mathbb {P}_{{\mathcal {M}}}(t_j|\sigma ) + \mathbb {P}_{{\mathcal {M}}}(t_k|\sigma ) \big ) \end{aligned}$$

where $i, j, k \in \{A, B, C\}$ with $i \ne j \ne k$. Now we can compute the differences in probabilities between $t_i$ and $t_j$ under the ${\mathcal {M}}{} \texttt {+UE}$ for any $i,j \in \{A, B, C\}$ such that $i \ne j$. We find

10 $$\begin{aligned}&\mathbb {P}_{{\mathcal {M}}{} \texttt {+UE}}(t_i|\alpha ,\beta ,\sigma ) - \mathbb {P}_{{\mathcal {M}}{} \texttt {+UE}}(t_j|\alpha ,\beta ,\sigma ) \nonumber \\&= \alpha ^2 \beta \cdot \big ( \mathbb {P}_{{\mathcal {M}}}(x_i | \sigma ) - \mathbb {P}_{{\mathcal {M}}}(x_j | \sigma ) \big ) \nonumber \\&\quad + \alpha (1 - \alpha ) (1 - \beta ) \cdot \big ( \mathbb {P}_{{\mathcal {M}}}(x_j | \sigma ) - \mathbb {P}_{{\mathcal {M}}}(x_i | \sigma ) \big ) \nonumber \\&\quad + (1 - \alpha ) (1 - \beta )^2 \cdot \big ( \mathbb {P}_{{\mathcal {M}}}(t_i | \sigma ) - \mathbb {P}_{{\mathcal {M}}}(t_j | \sigma ) \big ) \nonumber \\&\quad + \alpha \beta (1 - \beta ) \cdot \big ( \mathbb {P}_{{\mathcal {M}}}(t_j | \sigma ) - \mathbb {P}_{{\mathcal {M}}}(t_i | \sigma ) \big ) \nonumber \\&= \big ( \alpha ^2 \beta - \alpha (1-\alpha )(1 - \beta ) \big ) \cdot \big ( \mathbb {P}_{{\mathcal {M}}}(x_i | \sigma ) - \mathbb {P}_{{\mathcal {M}}}(x_j | \sigma ) \big ) \nonumber \\&\quad + \big ( (1 - \alpha )(1 - \beta )^2 - \alpha \beta (1 - \beta ) \big ) \cdot \big ( \mathbb {P}_{{\mathcal {M}}}(t_i | \sigma ) - \mathbb {P}_{{\mathcal {M}}}(t_j | \sigma ) \big ) \nonumber \\&= \alpha \big ( 1 - (\alpha + \beta ) \big ) \cdot \big ( \mathbb {P}_{{\mathcal {M}}}(x_j | \sigma ) - \mathbb {P}_{{\mathcal {M}}}(x_i | \sigma ) \big ) \nonumber \\&\quad + (1 - \beta ) \big ( 1 - (\alpha + \beta ) \big ) \cdot \big ( \mathbb {P}_{{\mathcal {M}}}(t_i | \sigma ) - \mathbb {P}_{{\mathcal {M}}}(t_j | \sigma ) \big ) \end{aligned}$$

Assuming that either condition (1) $\alpha = 0$ or condition (2) $\alpha + \beta \ne 1$ and $\mathbb {P}_{{\mathcal {M}}}(x_i | \sigma ) = \mathbb {P}_{{\mathcal {M}}}(x_j | \sigma )$, this quantity is zero if $\mathbb {P}_{{\mathcal {M}}}(t_i|\sigma ) = \mathbb {P}_{{\mathcal {M}}}(t_j|\sigma )$ and is greater than zero if $\mathbb {P}_{{\mathcal {M}}}(t_i|\sigma ) > \mathbb {P}_{{\mathcal {M}}}(t_j|\sigma )$. Because there are no anomalous triplets under ${\mathcal {M}}$, the former will be the case if $\sigma$ is non-binary; the latter will be the case if $\sigma = t_i$. It follows there are no anomalous triplets under the ${\mathcal {M}}{} \texttt {+UE}$ model, provided one of the two conditions hold. If these conditions do not hold and $\sigma \ne t_j$, triplet $t_j$ is anomalous when

11 $$\begin{aligned} \frac{\alpha }{(1 - \beta )} \cdot \big ( \mathbb {P}_{{\mathcal {M}}}(x_i | \sigma ) - \mathbb {P}_{{\mathcal {M}}}(x_j | \sigma ) \big ) - \big ( \mathbb {P}_{{\mathcal {M}}}(t_i | \sigma ) - \mathbb {P}_{{\mathcal {M}}}(t_j | \sigma ) \big ) > 0 \end{aligned}$$

for $i,j \in \{A,B,C\}$ such that $i \ne j$. $\square$

The result above extends easily to the case with unbiased missing values, in addition to unbiased error (see Corollary 1).

### Corollary 2

If there are no anomalous triplets under model ${\mathcal {M}}$, then there are no anomalous triplets under the ${\mathcal {M}}{} \texttt {+UEM}$ model, assuming that one of the two conditions: (1) $\alpha = 0$ or (2) $\alpha + \beta \ne 1$ and $\mathbb {P}_{{\mathcal {M}}}(100|\sigma ) = \mathbb {P}_{{\mathcal {M}}}(010|\sigma ) = \mathbb {P}_{{\mathcal {M}}}(001|\sigma )$. Otherwise, there can be anomalous triplets under the ${\mathcal {M}}{} \texttt {+UEM}$ model.

This concludes the derivation of the main result of this section (Theorem 2). Before moving onto phylogeny estimation, we consider whether anomalous triplets should be expected in the context of tumor phylogenetics, so setting ${\mathcal {M}}$ to be the $\texttt {IS}$ model. As previously mentioned, in the simulations performed by Kizilkale et al. [24], the largest values of $\alpha$ and $\beta$ were 0.001 and 0.2, respectively. This would put $\alpha /(1 - \beta ) = 0.00125$ in Eq. 11, so $\mathbb {P}_{{\mathcal {M}}}(x_i | \sigma ) - \mathbb {P}_{{\mathcal {M}}}(x_j | \sigma )$ would need to be 800 times greater than $\mathbb {P}_{{\mathcal {M}}}(t_i | \sigma ) - \mathbb {P}_{{\mathcal {M}}}(t_j | \sigma )$ for $t_j$ to be anomalous under the ${\mathcal {M}}{} \texttt {+UE}$ model (note the terms for missing values will cancel out in Eq. 11). Although this seems drastic, it could occur when $\sigma$ is created by restricting a larger model tree to a subset three leaves. Consider the cell lineage tree in Fig. 1 but with 1000 additional cells added between cell 9 and cell 10, and suppose that we sample cells $\{1, 4, 10\}$, so the resulting sub-model, $\sigma$ has rooted topology $t_{10} = 10|1, 4$. This submodel defines the following probability distribution on mutations, assuming mutations occur on all non-fake edges with equal probability. For the variant but triplet-uninformative patterns, we have $\mathbb {P}_{\texttt {IS}}(x_1|\sigma ) = 0$, $\mathbb {P}_{\texttt {IS}}(x_4|\sigma ) = 1/1012$, and $\mathbb {P}_{\texttt {IS}}(x_{10}| \sigma ) = 1009/1012$, and for triplet-informative patterns, we have $\mathbb {P}_{\texttt {IS}}(t_1|\sigma ) = 0$, $\mathbb {P}_{\texttt {IS}}(t_4|\sigma ) = 0$, and $\mathbb {P}_{\texttt {IS}}(t_{10}|\sigma ) = 1/1012$. Now looking at Eq. 11, we find that triplets $t_1$ and $t_4$ are anomalous under the IS + UEM model. Based on this analysis, we conjecture that triplets are less robust to error than quartets when sampling cells from a larger cell lineage tree.

## Phylogeny estimation from quartets

Because there are no anomalous quartets under the $\texttt {IS+UEM}$ model under reasonable assumptions (i.e., $\alpha + \beta \ne 1$), we now consider the utility of quartet-based methods for estimating cell lineage trees from mutation data. By quartet-based methods, we mean heuristics for the Maximum Quartet Support Supertree (MQSS) problem ( [4]; also see Section 7.7 in [27]).

### Definition 4

(Maximum Quartet Support Supertrees) The MQSS problem is defined by

Input: A set of unrooted trees ${\mathcal {P}} = \{T_1, T_2, \dots , T_k\}$, with tree $T_i$ on leaf label set $X_i$

Output: A unrooted, binary tree *B* on leaf label set $\cup _{i=1}^k X_i$ that maximizes $QS_D(B) = \sum _{q \in Q(B)} w_{{\mathcal {P}}}(q)$, where $w_{\mathcal {P}}(q)$ is the number of input trees in ${\mathcal {P}}$ that display *q*

When all input trees are on the same leaf label set, MQSS becomes weighted quartet consensus (WQC). An optimal solution to WQC is a consistent estimator of the unrooted species tree topology under the MSC model (Theorem 2 in [12]). Although MQSS and WQC are NP-hard [28, 29], fast and accurate heuristics have been developed, with the most well-known being ASTRAL [12]. Since version 2 [13, 14], ASTRAL allows the input trees to be incomplete and is statistically consistent under the MSC model, provided some assumptions on missing data (see [30] for details).

**Fig. 3:**
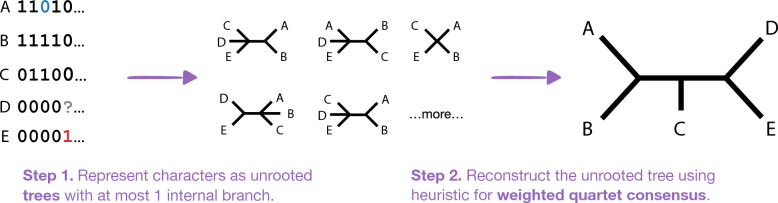
This figure shows a mutation matrix with one false negative (in blue), one false positive (in red), and one missing entry. The first step indicates how each mutation (column in the matrix) corresponds to an unrooted tree with at most one internal branch. The second step is to estimate the phylogeny by applying a quartet-based method. This approach is motivated by there being no anomalous quartets under the $\texttt {IS+UEM}$ model, assuming $\alpha + \beta \ne 1$ (Theorem 1)

There are two notable differences when using quartet-based methods to reconstruct cell lineage trees, rather than species trees. The first difference is that input are mutations rather than unrooted (gene) trees. This issue was addressed by Springer et al. [31], who treat mutations as unrooted trees with at most one internal branch (Fig. 3). Given this transformation of the input, it is possible to run ASTRAL and other quartet-based methods on mutation data. The second difference is that ASTRAL outputs a binary tree. Our next result suggests that MQSS/WQC are useful problem formulations, even when data are generated from non-binary trees.

### Theorem 3

Let $\sigma$ be a rooted tree with at least one internal branch when unrooted (so it can be non-binary), and let *D* be an $n \times k$ mutation matrix generated under the $\texttt {IS+UEM}$ model given $(\alpha , \beta , \gamma , \sigma )$ with $\alpha + \beta \ne 1$ and $0 \le \alpha , \beta , \gamma < 1$. Then, an optimal solution to MQSS given *D* is a consistent estimator of $u(\sigma )$ under the $\texttt{IS+UEM}$ model, with tree error defined as the number of false negative branches. If in addition $0< \alpha , \beta , < 1$, ASTRAL given *D* is statistically consistent under the $\texttt {IS+UEM}$ model, with tree error defined as the number of false negative branches.

The first statement above follows from Theorem 1 and Lemma 6. The second statement follows from Theorem 1, Corollary 3, and the observation that every complete mutation pattern is possible under the $\texttt {IS+UEM}$ model when $0< \alpha , \beta < 1$.

### Lemma 6

Let $\sigma$ be a rooted tree with at least one internal branch when unrooted (so it can be non-binary), and let *D* be an $n \times k$ mutation matrix generated under model ${\mathcal {A}}$ given $\sigma$. If there are no anomalous quartets under ${\mathcal {A}}$, an optimal solution to MQSS given *D* is a consistent estimator of $u(\sigma )$ under model ${\mathcal {A}}$, with tree error defined as the number of false negative branches.

### Proof

Let *B* be an unrooted, binary tree on the same label set as $u(\sigma )$. The number of false negative branches between *B* and $u(\sigma )$ is zero if *B* is a refinement of $u(\sigma )$, meaning that *B* can be obtained from $u(\sigma )$ in a sequence of refinement operations (this sequence has length zero if $\sigma$ is binary). Thus, to prove consistency with tree error defined as the number of false negative branches, we revise Definition 1 to say that for any $\epsilon > 0$, there exists a constant $K > 0$ such that when *D* contains at least *K* mutations, an optimal solution to MQSS given *D* is a refinement of $u(\sigma )$ with probability at least $1 - \epsilon$. The remainder of the proof follows from Lemma 7. $\square$

### Lemma 7

Suppose the conditions of Lemma 6 hold. Let $L(\sigma )$ be the label set of $\sigma$, and let *B* and *T* be unrooted, binary trees on $L(\sigma )$. Suppose that *B* is a refinement of $u(\sigma )$ and that *T* is NOT. Then, for any pair *B* and *T* and for any $\epsilon > 0$, there exists a constant $K > 0$ such that when *D* contains at least *K* mutations, $QS_D(B) > QS_D(T)$ with probability at least $1 - \epsilon$.

### Proof

To begin, we restate the inequality as

12 $$\begin{aligned} QS_D(B) - QS_D(T) = \sum _{S \in {\mathcal {X}}} w_D(B|_S) - \sum _{S \in {\mathcal {X}}} w_D(T|_S) > 0 \end{aligned}$$

where ${\mathcal {X}}$ is the set of all possible subsets of four elements from $L(\sigma )$ and $w_D(q)$ is the number of mutations in *D* that imply quartet *q*.

**Claim 1:** First, we claim that as $k \rightarrow \infty$, $w_D/k$ converges to its expectation $F^*$ under model ${\mathcal {A}}$ given $\sigma$ with probability 1. Claim 1 holds by the strong law of large numbers, as noted in the proofs of consistency for quartet and triplet-based methods under the $\texttt {MSC}$ model (see [20] for an example). Let $q_1^S$, $q_2^S$, $q_3^S$ denote the three possible quartets on *S*. Then, we can re-state claim 1 as follows. As $k \rightarrow \infty$, $w_D(q_i^S)/k$ converges to $F^*(q_i^S) = \mathbb {P}_{{\mathcal {A}}}(q_i^S | \sigma )$ with probability 1 for all $i \in \{1, 2, 3\}$ and for all $S \in {\mathcal {X}}$.

**Claim 2:** Second, we claim there exists a $\delta$ such that whenever $\Vert w_D/k - F_*\Vert _{\infty } < \delta$, Eq. 12 holds. We show claim 2 is true for $\delta = \pi / 2 |{\mathcal {X}}|$, where

13 $$\begin{aligned} \pi = \min _{>0,S \in {\mathcal {X}}} \{ |F^*(q_1^S) - F^*(q_2^S)|, |F^*(q_1^S) - F^*(q_3^S)|, |F^*(q_2^S) - F^*(q_3^S) | \}. \end{aligned}$$

There are three cases to consider.

*Case 1:* Let ${\mathcal {W}} \subset {\mathcal {X}}$ include all $S \in {\mathcal {X}}$ such that $B|_S$ and $T|_S$ are the same quartet. Then, $w_D(B|_S) = w_D(T|_S)$ for all $S \in {\mathcal {W}}$, giving us

14 $$\begin{aligned} \sum _{S \in {\mathcal {W}}} \bigg ( \frac{w_D(B|_S)}{k} - \frac{w_D(T|_S)}{k} \bigg ) = 0. \end{aligned}$$

Now suppose that *B* and *T* restricted to *S* display different quartets, denoted $q_1^S$ and $q_2^S$, respectively. If $q_2^S \in Q(u(\sigma))$, it must also be in *Q*(*B*) as $Q(u(\sigma)) \subseteq Q(B)$. Therefore, $q_2^S \notin Q(u(\sigma))$. This gives us two additional cases to consider.

*Case 2:* Let ${\mathcal {Y}} \subset {\mathcal {X}}$ include all $S \in {\mathcal {X}}$ such that $B|_S$ and $T|_S$ are different quartets, denoted $q_1^S$ and $q_2^S$, respectively, with $q_1^S \in Q(u(\sigma))$ and $q_2^S \notin Q(u(\sigma))$. Then, for all $S \in {\mathcal {Y}}$, $F^*(q_1^S) > F^*(q_2^S)$ and $F^*(q_1^S) > F^*(q_3^S)$ because there are no anomalous quartets under ${\mathcal {A}}$ (Definition 2). Therefore, whenever $\Vert w_D/k - F_*\Vert _{\infty } < \pi /2$, $w_D(B|_S) > w_D(T|_S)$ for all $S \in {\mathcal {Y}}$, giving us

15 $$\begin{aligned} \sum _{S \in {\mathcal {Y}}} \bigg ( \frac{w_D(B|_S)}{k} - \frac{w_D(T|_S)}{k} \bigg ) > 0. \end{aligned}$$

*Case 3 (only needed for non-binary* $\sigma$): Let ${\mathcal {Z}} \subset {\mathcal {X}}$ include all $S \in {\mathcal {X}}$ such that $B|_S$ and $T|_S$ are different quartets, denoted $q_1^S$ and $q_2^S$, respectively, with $q_1^S, q_2^S \notin Q(u(\sigma))$. By our assumptions on *B*, $\sigma|_S$ is a star. Then, because there are no anomalous quartets under ${\mathcal {A}}$ (Definition 2), $F^*(q_1^S) = F^*(q_2^S) = F^*(q_3^S)$. However, even as $k \rightarrow \infty$, we are not guaranteed to get an exact equality $w_D(q_1^S) = w_D(q_2^S) = w_D(q_3^S)$. Thus, we need to put an upper bound $\delta$ on $\Vert w_D/k - F_*\Vert _{\infty }$ so that Eq. 12 holds even when $w_D(B|_S) < w_D(T|_S)$ for all $S \in {\mathcal {Z}}$. This happens for $\delta = \pi / 2 |{\mathcal {X}}|$. When $\Vert w_D/k - F_*\Vert _{\infty } < \pi / 2 |{\mathcal {X}}|$, we have

16 $$\begin{aligned} \sum _{S \in {\mathcal {Z}}} \bigg | \frac{w_D(B|_S)}{k} - \frac{w_D(T|_S)}{k} \bigg |< |{\mathcal {Z}}| \cdot \frac{\pi }{|{\mathcal {X}}|}< |{\mathcal {Y}}| \cdot \bigg ( \pi - \frac{\pi }{|{\mathcal {X}}|} \bigg ) < \sum _{S \in {\mathcal {Y}}} \bigg ( \frac{w_D(B|_S)}{k} - \frac{w_D(T|_S)}{k} \bigg ) \end{aligned}$$

because $(|{\mathcal {Y}}| + |{\mathcal {Z}}|)/ |{\mathcal {X}}| \le 1$ and $1 \le |{\mathcal {Y}}|$. If not, either *T* is a refinement of $u(\sigma )$ or $u(\sigma )$ has no internal branches, contradicting our assumptions.

*Putting the cases together:* If $u(\sigma )$ is binary, then ${\mathcal {W}}|{\mathcal {Y}}$ is a partition ${\mathcal {X}}$, so we can combine equations 14 and 15 to get Eq. 12. If $u(\sigma )$ is non-binary, then ${\mathcal {W}}|{\mathcal {Y}}|{\mathcal {Z}}$ is a partition of ${\mathcal {X}}$, so we can combine Eqs. 14 and 16 to get Eq. 12.

Wrap up. By claim 1, for any $\epsilon > 0$, there exists a constant $K > 0$ such that when *D* contains at least *K* mutations, $\Vert w_D/k - F^*\Vert _{\infty } < \pi / 2 |{\mathcal {X}}|$ with probability at least $1 - \epsilon$. Then, by claim 2, for any $\epsilon > 0$, when *D* contains at least *K* mutations, $QS_D(B) > QS_D(T)$ with probability at least $1 - \epsilon$. $\square$

### Corollary 3

Suppose the conditions of Lemma 6 hold. If there are no anomalous quartets under model ${\mathcal {A}}$ and every complete mutation pattern occurs with non-zero probability under ${\mathcal {A}}$, then ASTRAL given *D* is statistically consistent under ${\mathcal {A}}$, with tree error defined as the number of false negative branches.

### Proof

ASTRAL solves MQSS exactly within a constrained version of the solution space, denoted $\Sigma$. Its algorithm has two main steps: first, form $\Sigma$ so that it contains all bipartitions induced by the input “trees” (i.e., mutations in *D*), and second find a solution *B* to MQSS such that $Bip(B) \subseteq \Sigma$. Because all complete mutation patterns have non-zero probability under the model ${\mathcal {A}}$, for any $\epsilon _1 > 0$, there exists a constant $K_1> 0$ such that when $k \ge K_1$, $\Sigma$ will contain all bipartitions induced by at least one refinement of $u(\sigma )$ with probability $1 - \epsilon _1$. By Lemma 7, for any $\epsilon _2 > 0$, there exists a constant $K_2 > 0$ such that when $k \ge K_2$, $QS_D(B) > QS_D(T)$ for any pair *B* and *T* of unrooted binary trees on $L(\sigma )$ such that *B* is a refinement of $u(\sigma )$ and *T* is NOT. Now let $\epsilon > 0$ and select $\epsilon _1, \epsilon _2 > 0$ such that $\epsilon _1 + \epsilon _2 < \epsilon$. Then, when *D* contains at least $\max \{K_1, K_2\}$ mutations, ASTRAL given *D* returns a refinement of $u(\sigma )$ with probability at least $(1 - \epsilon _1)(1-\epsilon _2) > (1 - \epsilon )$. It follows the number of false negative branches is zero with probability at least $1 - \epsilon$. $\square$

Lastly, we note that related results can be derived for triplets by viewing mutations as a rooted trees with at most one internal branch (Additional file 1: Fig. S2).

### Theorem 4

Suppose that the conditions of Theorem 3 hold but that $\alpha = 0$ (instead of $\alpha + \beta \ne 1$). Then, an optimal solution to Maximum Triplet Support Supertree (MTSS) problem given *D* is a consistent estimator of $\sigma$ under the $\texttt {IS+UEM}$ model, with tree error defined as the number of false negative branches.

This result follows from Theorem 2 and Lemma 6 but replacing “quartet” with “triplet” and “rooted” with “unrooted”. Code for transforming ordered 2-state characters into rooted trees (as shown in Additional file 1: Fig. S2) is available as part of Dollo-CDP [32]; see the − k option on Github: https://github.com/molloy-lab/Dollo-CDP. These “rooted trees” can be given as input to quartet-based methods, like ASTRAL or ASTER [18], which will effectively ignore the root (note that any “tree” on fewer than four cells must be removed prior to running ASTRAL).

## Discussion

Quartet-based approaches have garnered much success for estimating species phylogenies under the Multi-Species Coalescent [12–14]. Here, we considered their application for estimating cell lineage trees, focusing on two differences between estimating cell lineage trees compared to species trees. First, errors and missing values can arise from single-cell sequencing and thus are typically modeled. Second, the model cell lineage tree may be highly unresolved because tumors evolve clonally. To address these issues, we first show that there are no anomalous quartets under the infinite sites ($\texttt {IS}$) plus unbiased error and missingness ($\texttt {UEM}$) model, which is widely used in tumor phylogenetics (this is an *identifiability result*). We then show that under the $\texttt {IS+UEM}$ model, an optimal solution to the Maximum Quartet Support Supertree (MQSS) problem is a refinement of the model cell lineage tree (this is a *consistency result* when tree error is defined as the number of false negative branches). Lastly, we consider the case of triplets, showing that there can be anomalous triplets when the probability of false positive errors is greater than zero. Our result suggests that quartets may be more robust to error than triplets when reconstructing cell lineage trees.

Overall, our results suggest the potential of quartet-based methods for reconstructing trees from noisy mutation data, provided that the tree can be rooted and that false positive branches in the output tree can be effectively handled. The former is often doable because the tree can be rooted on the edge incident to the healthy cell with no mutations. The latter is related to mapping mutations onto branches in the cell lineage tree (see [33]) as well as identifying which cells are members of the same clone or subclone (see [34]). These tasks are also relevant to likelihood-based methods designed for cell lineage tree reconstruction, as such methods also return binary trees. Examples include ScisTree [23], SiFit [35], and CellPhy [33] (note that of these methods ScisTree makes the $\texttt {IS}$ assumption but the other two do not).

In general, likelihood-based methods require explicitly estimating numeric parameters, like $\alpha$ and $\beta$, as well as exploring the space of cell lineage trees, which grows exponentially in the numbers of cells. In contrast, quartet-based approaches allow for error implicitly (without explicit estimation of $\alpha$ and $\beta$) and are often based on algorithmic techniques, like divide-and-conquer, that are quite fast in practice. That being said, quartet-based methods have been designed for species phylogenetics, where the number of leaves (species) is typically much less than the number of gene trees or characters. In tumor phylogenetics, the number of leaves (cells) can be much greater than than characters. This will likely to have consequences for runtime and accuracy (just consider that our consistency guarantees is in the limit of infinite mutations). Corollary 3 sheds light on a potential issue when using ASTRAL, namely the construction of the constrained solution space $\Sigma$ may not be very successful for mutation data, especially if the number of mutations is small. However, there are other high quality heuristics for MQSS, including wQMC [36–38], wQFM [16, 39], and TREE-QMC [17]. Moreover, even when the number of characters is small compared to the number of cells, the underlying model tree is likely to be highly unresolved. In this case, sampling different cells around the same branch may be a means of providing more data for estimation (this observation has already been leveraged by Kizilkale et al. [24]).

Although quartet-based methods, as presented here, may be robust to noise, they fail to address doublets and copy number aberrations (CNAs), which also challenge cell lineage tree reconstruction. A doublet is a sequencing artifact where data provided for a single cell is really a mixture of two cells. This “hybrid” cell challenges the notion of tree-like evolution, motivating the development of methods for correcting doublets [40]. If doublets can be effectively corrected, then their impact on quartet-based methods would be minimal. Alternatively, quartets may be useful for detecting doublets. CNAs include duplications and losses of large sections of chromosomes (see review on methods for detecting CNAs by Mallory et al. [41]). CNA losses, in particular, have motivated the development of many new methods for reconstructing tumor phylogenies [42–44]. Some of these methods view CNA losses as false negatives (although these false negatives will be biased towards particular cells and mutations). In contrast, SCARLET [44] reconstructs a CNA tree and then uses it to constrain phylogeny reconstruction with the mutation data. Constraints have also been leveraged in species phylogenetics, including with ASTRAL [45]. Thus, the output of quartet-based methods could similarly be forced to obey the constraints of a CNA tree. To summarize, there are practical limitations to quartet-based methods for tumor phylogenetics, several of which apply to existing methods that do not handle CNAs and doublets, for example.

Looking beyond cell lineage tree reconstruction, our results generalize beyond the $\texttt {IS}$ model to any model of 2-state character evolution for which there are no anomalous quartets or triplets. A consequence of our study is that quartet-based methods, like ASTRAL, are consistent under the $\texttt {IS+nWF}$ model, even when unbiased errors and missing values are introduced. This statement follows from combining Corollaries 1 and 3 with Theorem 1 in [48]. Thus, our work addresses an open question from [48] about the utility of such methods on imperfect data and gives a positive result for recent systematic studies leveraging quartet-based methods on retroelement insertion presence/absence patterns for placental mammals [51], bats [52], and birds [53]. Missing values, in particular, are prevalent in these data sets (e.g., the data set from [54] has 18% missing/ambiguous values). Future work should investigate this issue further, looking at error and missingness biased towards particular species or (orthologous) positions of the genome (related questions would also be of interest in the context of cell lineage tree estimation). Similarly, while unbiased errors (false positives and false negatives) may be appropriate for modeling sequencing error, it may not be appropriate in this other setting if error is biased towards particular species or genes when calling retroelement insertions. Lastly, species trees are typically assumed to be binary; however, there could be hard polytomies, in which case the model tree would be non-binary. Our results for consistency with error defined as the number of false negatives (Lemma 6 and Corollary 3) extend to the other models, like $\texttt {MSC}$, suggesting the utility of quartet-based methods in the case of hard polytomies.

### Supplementary Information

**Additional file 1.** Proofs of Lemmas 1, 3, and 5, supplemental figures S1–S2, and supplemental tables S1–S16.

## Data Availability

Not applicable.

## Abbreviations

CNA: Copy number abberation
IS: Infinite sites
MQSS: Maximum quartet support supertree
MSC: Multi-species coalescent
MTSS: Maximum triplet support supertree
nWF: Neutral Wright-Fisher
UEM: Unbiased error and missingness

## References

[1] Lim B, Lin Y, Navin N (2020). Advancing cancer research and medicine with single-cell genomics. Cancer Cell.

[2] Jahn K, Kuipers J, Beerenwinkel N (2016). Tree inference for single-cell data. Genome Biol.

[3] Schwartz R, Schäffer AA (2017). The evolution of tumour phylogenetics: principles and practice. Nat Rev Genet.

[4] Wilkinson M, Cotton JA, Creevey C, Eulenstein O, Harris SR, Lapointe F-J, Levasseur C, Mcinerney JO, Pisani D, Thorley JL (2005). The shape of supertrees to come: tree shape related properties of fourteen supertree methods. Syst Biol.

[5] Pamilo P, Nei M (1988). Relationships between gene trees and species trees. Mol Biol Evol.

[6] Rannala B, Yang Z (2003). Bayes estimation of species divergence times and ancestral population sizes using DNA sequences from multiple loci. Genetics.

[7] Allman ES, Degnan JH, Rhodes JA (2011). Identifying the rooted species tree from the distribution of unrooted gene trees under the coalescent. J Math Biol.

[8] Degnan JH (2013). Anomalous unrooted gene trees. Syst Biol.

[9] Kubatko LS, Degnan JH (2007). Inconsistency of phylogenetic estimates from concatenated data under coalescence. Syst Biol.

[10] Roch S, Steel M (2015). Likelihood-based tree reconstruction on a concatenation of aligned sequence data sets can be statistically inconsistent. Theor Popul Biol.

[11] Larget BR, Kotha SK, Dewey CN, Ané C (2010). BUCKy: gene tree/species tree reconciliation with Bayesian concordance analysis. Bioinformatics.

[12] Mirarab S, Reaz R, Bayzid MS, Zimmermann T, Swenson MS, Warnow T (2014). ASTRAL: genome-scale coalescent-based species tree estimation. Bioinformatics.

[13] Mirarab S, Warnow T (2015). ASTRAL-II: coalescent-based species tree estimation with many hundreds of taxa and thousands of genes. Bioinformatics.

[14] Zhang C, Rabiee M, Sayyari E, Mirarab S (2018). ASTRAL-III: polynomial time species tree reconstruction from partially resolved gene trees. BMC Bioinf.

[15] Dibaeinia P, Tabe-Bordbar S, Warnow T (2021). FASTRAL: improving scalability of phylogenomic analysis. Bioinformatics.

[16] Mahbub M, Wahab Z, Reaz R, Rahman MS, Bayzid MS (2021). wQFM: highly accurate genome-scale species tree estimation from weighted quartets. Bioinformatics.

[17] Han Y, Molloy EK (2023). Improving quartet graph construction for scalable and accurate species tree estimation from gene trees. Genome Res.

[18] Zhang C, Mirarab S (2022). Weighting by gene tree uncertainty improves accuracy of quartet-based species trees. Mol Biol Evol.

[19] Degnan JH, Rosenberg NA (2006). Discordance of species trees with their most likely gene trees. PLoS Genet.

[20] Liu L, Yu L, Edwards SV (2010). A maximum pseudo-likelihood approach for estimating species trees under the coalescent model. BMC Evol Biol.

[21] Islam M, Sarker K, Das T, Reaz R, Bayzid MS (2020). STELAR: a statistically consistent coalescent-based species tree estimation method by maximizing triplet consistency. BMC Genomics.

[22] Ross EM, Markowetz F (2016). OncoNEM: inferring tumor evolution from single-cell sequencing data. Genome Biol.

[23] Wu Y (2019). Accurate and efficient cell lineage tree inference from noisy single cell data: the maximum likelihood perfect phylogeny approach. Bioinformatics.

[24] Kizilkale C, Mehrabadi FR, Azer ES, Pérez-Guijarro E, Marie KL, Lee MP, Day C-P, Merlino G, Ergün F, Buluç A, Sahinalp SC, Malikić S (2022). Fast intratumor heterogeneity inference from single-cell sequencing data. Nat Comput Sci.

[25] Fisher RA (1923). On the dominance ratio. Proc R Soc Edinb.

[26] Wright S (1931). Evolution in mendelian populations. Genetics.

[27] Warnow T (2017). Computational phylogenetics: an introduction to designing methods for phylogeny estimation.

[28] Jiang T, Kearney P, Li M (2001). A polynomial time approximation scheme for inferring evolutionary trees from quartet topologies and its application. SIAM J Comput.

[29] Lafond M, Scornavacca C (2019). On the weighted quartet consensus problem. Theor Comput Sci.

[30] Nute M, Chou J, Molloy EK, Warnow T (2018). The performance of coalescent-based species tree estimation methods under models of missing data. BMC Genomics.

[31] Springer MS, Molloy EK, Sloan DB, Simmons MP, Gatesy J (2019). ILS-aware analysis of low-homoplasy retroelement insertions: inference of species trees and introgression using quartets. J Hered.

[32] Dai J, Rubel T, Han Y, Molloy EK. Leveraging Constraints plus dynamic programming for the large dollo parsimony problem. In: Belazzougui D, Ouangraoua A, editors. 23rd International Workshop on Algorithms in Bioinformatics (WABI 2023). Leibniz International Proceedings in Informatics (LIPIcs), vol. 273. Schloss Dagstuhl–Leibniz-Zentrum für Informatik, Dagstuhl, Germany. 2023. pp. 5–1523. 10.4230/LIPIcs.WABI.2023.5

[33] Kozlov A, Alves JM, Stamatakis A, Posada D (2022). Cell Phy: accurate and fast probabilistic inference of single-cell phylogenies from scDNA-seq data. Genome Biol.

[34] Zafar H, Navin N, Chen K, Nakhleh L (2019). SiCloneFit: Bayesian inference of population structure, genotype, and phylogeny of tumor clones from single-cell genome sequencing data. Genome Res.

[35] Zafar H, Tzen A, Navin N, Chen K, Nakhleh L (2017). SiFit: inferring tumor trees from single-cell sequencing data under finite-sites models. Genome Biol.

[36] Snir S, Rao S (2010). Quartets MaxCut: a divide and conquer quartets algorithm. IEEE/ACM Trans on Comput Biol Bioinf.

[37] Snir S, Rao S (2012). Quartet MaxCut: a fast algorithm for amalgamating quartet trees. Mol Phylogenet Evol.

[38] Avni E, Cohen R, Snir S (2014). Weighted quartets phylogenetics. Syst Biol.

[39] Reaz R, Bayzid MS, Rahman MS (2014). Accurate phylogenetic tree reconstruction from quartets: a heuristic approach. PLoS ONE.

[40] Weber LL, Sashittal P, El-Kebir M (2021). doubletD: detecting doublets in single-cell DNA sequencing data. Bioinformatics.

[41] Mallory XF, Edrisi M, Navin N, Nakhleh L (2020). Methods for copy number aberration detection from single-cell DNA-sequencing data. Genome Biol.

[42] El-Kebir M (2018). SPhyR: tumor phylogeny estimation from single-cell sequencing data under loss and error. Bioinformatics.

[43] Malikic S, Mehrabadi FR, Ciccolella S, Rahman MK, Ricketts C, Haghshenas E, Seidman D, Hach F, Hajirasouliha I, Sahinalp SC (2019). PhISCS: a combinatorial approach for subperfect tumor phylogeny reconstruction via integrative use of single-cell and bulk sequencing data. Genome Res.

[44] Satas G, Zaccaria S, Mon G, Raphael BJ (2020). SCARLET: single-cell tumor phylogeny inference with copy-number constrained mutation losses. Cell Syst.

[45] Rabiee M, Mirarab S (2020). Forcing external constraints on tree inference using astral. BMC Genomics.

[46] Hudson RR (2002). Generating samples under a Wright-Fisher neutral model of genetic variation. Bioinformatics.

[47] Kuritzin A, Kischka T, Schmitz J, Churakov G (2016). Incomplete lineage sorting and hybridization statistics for large-scale retroposon insertion data. PLOS Comput Biol.

[48] Molloy EK, Gatesy J, Springer MS (2021). Theoretical and practical considerations when using retroelement insertions to estimate species trees in the anomaly zone. Syst Biol.

[49] Mendes FK, Hahn MW (2017). Why concatenation fails near the anomaly zone. Syst Biol.

[50] Springer MS, Gatesy J (2016). The gene tree delusion. Mol Phylogenet Evol.

[51] Doronina L, Hughes GM, Moreno-Santillan D, Lawless C, Lonergan T, Ryan L, Jebb D, Kirilenko BM, Korstian JM, Dávalos LM, Vernes SC, Myers EW, Teeling EC, Hiller M, Jermiin LS, Schmitz J, Springer MS, Ray DA (2022). Contradictory phylogenetic signals in the laurasiatheria anomaly zone. Genes.

[52] Korstian J, Paulat N, Platt R, Stevens R, Ray D (2022). Sine-based phylogenomics reveal extensive introgression and incomplete lineage sorting in myotis. Genes.

[53] Gatesy J, Springer MS (2022). Phylogenomic coalescent analyses of avian retroelements infer zero-length branches at the base of neoaves, emergent support for controversial clades, and ancient introgressive hybridization in afroaves. Genes.

[54] Cloutier A, Sackton TB, Grayson P, Clamp M, Baker AJ, Edwards SV (2019). Whole-genome analyses resolve the phylogeny of flightless birds (Palaeognathae) in the presence of an empirical anomaly zone. Syst Biol.

